# Family Affluence Based Inequality in Oral Health-Related Quality of Life in a Population of Lithuanian Adolescents

**DOI:** 10.3390/ijerph16122106

**Published:** 2019-06-14

**Authors:** Apolinaras Zaborskis, Aistė Kavaliauskienė, Antanas Šidlauskas

**Affiliations:** 1 Department of Preventive Medicine, Faculty of Public Health, Medical Academy, Lithuanian University of Health Sciences, Tilžės street 18, LT-47181 Kaunas, Lithuania; 2 Department of Orthodontics, Faculty of Odontology, Medical Academy, Lithuanian University of Health Sciences, A.Lukšos-Daumanto street 6, LT-50106 Kaunas, Lithuania; aiste.kavaliauskiene@lsmuni.lt (A.K.); antanas.sidlauskas@lsmuni.lt (A.Š.)

**Keywords:** social inequality in health, oral health-related quality of life, child perceptions questionnaire, malocclusion, family affluence, adolescents, Lithuania

## Abstract

Background: The social inequalities in oral health have had increasing attention in recent years. The present study aimed to explore the impact of family affluence on Oral Health-Related Quality of Life (OHRQoL) among Lithuanian adolescents aged 11–18 years. Methods: The cross-sectional, population-based study included a representative sample of 881 adolescents aged 11–18 years (mean = 15.55; SD = 1.51) randomly selected from 20 schools in Lithuania. The schoolchildren completed questionnaires to evaluate their OHRQoL using a Lithuanian version of the Child Perceptions Questionnaire (CPQ). The adolescents’ family affluence was indirectly assessed by inquiring whether they possessed various modern life items. In dental examination, the severity of malocclusion was predetermined by the Index of Complexity, Outcome, and Need (ICON). The relationship among variables was examined employing the negative binomial regression and the path analysis. Results: The sum score of CPQ as a whole and the sum scores of all four domains were significantly associated with family affluence, indicating higher OHRQoL among adolescents from more affluent families. The severity of malocclusion had a significant association with emotional and social well-being domains of OHRQoL only. Conclusion: This study evidences the family affluence based inequality in OHRQoL among Lithuanian adolescents.

## 1. Introduction

In Lithuania and internationally, malocclusion is a major contributor to oral health among adolescents [1,2,3]. Malocclusion causes a lot of trouble, but pain is not one of them. It can result also disturbances of oral function such as mastication, swallowing, and speech, as well as can cause psychosocial problems related to impaired dento-facial aesthetics which exert an adverse impact on the Oral Health-Related Quality of Life (OHRQoL) [4,5,6]. Concern with the impact of malocclusion on OHRQoL has grown considerably in recent years as a consequence of increased patient awareness of this condition, as well as of heightened expectations regarding orthodontic treatment opportunities [7]. The importance of OHRQoL is specifically relevant for adolescents, because they are particularly sensitive to a variety of impacts, such as appearance, that may affect their well-being and influence their social skills and education [8,9]. It has been suggested that the OHRQoL should be measured in oral health surveys for children and adolescents [10].

Previous investigations have shown that a more unfavorable socioeconomic status (SES) generally translates to inferior oral health among children and adolescents [11,12]. The existence of social inequality may, therefore, also have impact upon the treatment of malocclusion. Most malocclusion cases are still not treated properly due to the lack of awareness of young patients and their parents about this condition, the inadequacy of dental resources, inaccessibility to dental services, the lack of a dental workforce, and many factors related to SES. Malocclusion treatment is often expensive and can be impossible for disadvantaged populations. It has also been demonstrated that SES resulted in poorer OHRQoL [13,14,15,16]. For instance, in the study of Brazilian schoolchildren aged 12 years, after adjusting for potential confounders, family income and mothers’ education showed a statistically significant association with all health domains of the OHRQoL [17].

In general, OHRQoL is measured by instruments that have been validated for this purpose [18]. Among these instruments, the Child Perceptions Questionnaire (CPQ_11–14_), developed for children aged 11–14 years by a group of Canadian researchers [19], has gained the highest popularity. It describes the following domains of OHRQoL: oral symptoms (OS), functional limitations (FL), emotional well-being (EWB), and social well-being (SWB) [19]. It is important to consider that clinical and social determinants may have different impacts on domains of OHRQoL. Therefore, the evaluation of OHRQoL by its domains (OS, FL, EWB, SWB) allows for a more detailed analysis of SES determinants. However, the impact of adolescent family affluence on each domain has not yet been adequately explored, and the currently available results in this field of research are controversial [17].

This study also intends to examine the impact of malocclusion on OHRQoL. Though, the objective quantitative assessment of malocclusion was necessary. The prevalence and severity of the various features of malocclusion can be assessed using orthodontic indexes. The index of orthodontic treatment need (IOTN) and index of complexity, outcome and need (ICON) are the most commonly used indexes in epidemiological studies [20,21,22]. Our choice of ICON in this study was based on its advantage over other orthodontic indexes. ICON incorporates the esthetic score into an integral part of the five assessment components. Even more, what is important for epidemiological studies, the components can be measured directly on patients (no need to have dental casts). The present study aims to explore the impact of family affluence on OHRQoL among Lithuanian adolescents aged 11–18 years. The hypotheses tested in this study were: (1) higher family affluence is associated with better adolescent OHRQoL; (2) family affluence influences each CPQ domain specifically; (3) family affluence has a greater effect on OHRQoL than on malocclusion.

## 2. Materials and Methods 

### 2.1. Ethical Statement

The ethical approval for the examination of schoolchildren was issued by the Kaunas Regional Biomedical Research Ethics Committee on 27 November 2012 (No. BE-2-47). Written informed consent for child’s examination was obtained from both parents of each child who participated in the study. Confidentiality and anonymity of participants was guaranteed.

### 2.2. Sample Size Calculation

The calculation of sample size was produced with software G*Power 3.1 (University of Dusseldorf, Dusseldorf, Germany) [23]. The procedure of calculation assumed a Poisson regression, considering 80% power, a confidence level of 5%, and a mean sum score ratio to be detected of at least 1.1. For each age group, the minimum number of participants required by these parameters was 260. Thus, since three age groups were studied, the total required sample size was estimated to be *n* = 3 × 260 = 780.

### 2.3. Study Design, Participants, and Data Collection 

This observational study had a cross-sectional design and targeted adolescents from 11 to 18 years old, who were divided into three age groups: 11–14, 15–16, and 17–18.

A random, two-stage sampling design was used to select the initial sample. Twenty public schools were randomly selected from the list of schools in Lithuania, and, in each school, classes from grades 6 to 11 were randomly selected. The initial sample (*n* = 1464) was almost twice as big as the estimated required value, because only half of the students were expected to participate in the study.

The principals of the selected schools were contacted introducing the study and discussing the most appropriate circumstances of the schoolchildren’s examination. An information letter was then sent to parents asking them for permission their child to be examined.

Both a questionnaire survey and dental examination were conducted to collect data. Under the supervision of a class teacher, schoolchildren completed anonymous self-administrated questionnaires in the classroom before their dental examination. Dental examinations were performed in school medical offices.

In the selected schools, 1160 students participated in the questionnaire survey, and 996 students completed the dental examination. Thus, the response rates for the questionnaire survey and examination were 80% and 68%, respectively.

### 2.4. Evaluation of Oral Health-Related Quality of Life

The Child Perception Questionnaire (CPQ), originally developed by Jokovic et al. [19], was used to evaluate OHRQoL. The Lithuanian version of this questionnaire was translated, revised, and validated by Kavaliauskiene et al. [24]. The instrument, which includes a modification of the item concerning oral pain, has presented good results for the assessment of psychometric properties among adolescents 11 to 18 years of age [24,25].

The whole CPQ consists of 37 items scored on a 5-point Likert scale ranging from 0 (“never”) to 4 (“every day or almost every day”) (Supplementary File S1). There are four subscales to describe OHRQoL domains: oral symptoms (OS, six items), functional limitations (FL, nine items), emotional well-being (EWB, nine items), and social well-being (SWB, 13 items). In the analysis, the scores for each item were added together to get a sum score of each domain, as well as of the whole CPQ. Note that higher sum scores refer to inferior OHRQoL.

### 2.5. Evaluation of Orthodontic Status

The dental examinations were performed according to the recommendations of the World Health Organization for epidemiological surveys [26]. Orthodontic examination was a part of the complex dental examination. All students were examined by one orthodontic specialist (A.K.) who was trained and graded in the reliability of assessing orthodontic status (U.K. Cardiff University School of Dentistry, 2012).

The Index of Complexity, Outcome, and Need (ICON) has been shown to be a valid measure of orthodontic status in epidemiological studies [21,22]. The index was recorded using the methodology by Richmond [27]. A single ICON score is based on five occlusal traits that are weighted and then added together: (1) an aesthetic component (AC) (weighted by 7); (2) upper arch crowding or spacing (weighted by 5); (3) crossbite (weighted by 5); (4) overbite or open bite (weighted by 4); and (5) left and right buccal segment antero-posterior relationship (weighted by 3). The AC consists of 10 color photographs showing front-view dentition graded from 1 (most attractive) to 10 (least attractive) which were used to identify and rate the participant (Supplementary File S2). A cut-off point of 43 was set to mark the unambiguous need for orthodontic treatment [27].

### 2.6. Evaluation of Family Affluence

The social position of adolescents was evaluated by their family affluence using Family Affluence Scale (FAS), which was specially designed for the questionnaire surveys among children and adolescents [28]. For this purpose, the adolescents were asked how many cars, home computers, child’s bedrooms, and travels with children on holiday their family had (Supplementary File S1). Based on respondent’s responses to these four items, a sum score was calculated. Then, a three-point ordinal variable FAS was compiled: 1 = low affluence (score 0–3); 2 = medium affluence (score 4–5); 3 = high affluence (score 6 and more) [28].

### 2.7. Statistical Analysis

The study data were analyzed using the SPSS statistical package supplemented with AMOS (version 21; IBM SPSS Inc, Chicago, IL, USA, 2012). Descriptive statistics were first estimated as the mean, standard deviation, median, interquartile range (IQR), and percentages, as appropriate. Categorical variables were tested by the Chi-square test and Z test with Bonferroni correction. *p*-values were obtained from two-sided statistical tests, and significance level was *p* ≤ 0.05.

The Kolmogorov–Smirnov test showed that the sum scores of the whole CPQ and its domains had a non-normal distribution. They were skewed in the direction of low values, and consequently, more-or-less followed a Poisson distribution. Thus, non-parametric methods of statistical data analysis were employed. For the same reason, the direct association of the sum scores of the CPQ with clinical and socio-demographic variables was analyzed using the Negative Binomial Regression (NBR), a modified Poisson regression model [29,30,31]. In the present analyses, the sum score of the whole CPQ or its domains was the dependent variable, and the ICON and socio-demographic variables were the independent variables. The strength of association between dependent and independent variables was measured using the Ratio of Sum Score Means (RSSM), which indicates how many times the mean value of a dependent variable increases when the value of an independent variable increases by one unit.

Finally, path analysis was used to examine the hypothesized causal relationships of OHRQoL with ICON, family affluence, gender, and age. These relationships were assumed to be unidirectional. Structural equation modelling was conducted to assess the final model using a maximum likelihood estimation method, given its applicability to non-normal data [32,33,34]. The final model provided standardized regression weights (*β*) which showed the strength of the relationship between two connected variables. The χ^2^ statistic was used to assess the magnitude of the discrepancy between the sample and fitted covariance matrices, where *p* > 0.05 indicated that the model and data were consistent. In addition, the root mean square error of approximation (RMSEA), the comparative fit index (CFI), and the incremental fit index (IFI) were employed to evaluate the model’s goodness-of-fit (RMSEA < 0.08, and CFI > 0.90, and IFI > 090 indicate an acceptable model fit; and RMSEA < 0.05, and CFI > 0.95 and IFI > 0.95 indicate a very good fit) [32]. Path analysis was performed using AMOS 21 (SPSS Inc., Chicago, IL, USA) [33].

## 3. Results

### 3.1. Descriptive Statistics and Correlations

The analysis included 881 students (40.9% male, 59.1% female), aged 11–18 (mean = 15.55; SD = 1.51), who participated both in the questionnaire survey and in dental examination. They provided complete data sets (CPQ and ICON sum scores, socio-demographic variables). Almost half (48.0%) were regarded as living in highly wealthy families. Descriptive statistics including absolute frequencies and percentages for categorical variables and means and standard deviations for continuous variables are presented in Table 1. In addition, medians and interquartile intervals are presented for the sum score of the whole CPQ and its domains, as the distribution deviated substantially from normal and was skewed in the direction of low values.

Spearman’s correlation coefficient values, shown in Table 2, indicate that the FAS sum score has a negative and significant relationship with the whole CPQ and its domains OS, FL, and EWB, but it has a weak relationship with the SWB domain, indicating that higher family affluence is related to a lower CPQ sum score or higher OHRQoL in corresponding domains. The FAS sum score is not related to the ICON sum score. The ICON sum score showed a positive and significant association with the sum score of the EWB and SWB domains only, although all four CPQ domains were moderately associated with each other.

### 3.2. Direct Influence of Malocclusion and Family Affluence on OHRQoL

The presence of a direct association between the sum scores of the CPQ and severity of malocclusion defined by ICON and family affluence was explored by employing multivariate NBR analysis (Table 3).

Like the correlation analysis, this analysis showed that the need for orthodontic treatment is significantly associated with the EWB and SWB domains, as well as with the CPQ as a whole. The assessments of the relationship (RSSM > 1) indicated that subjects who needed orthodontic treatment had higher sum scores, corresponding to inferior emotional and subjective well-being domains of OHRQoL. Higher family affluence was associated with a lower sum score in all domains (RSSM < 1); however, a significant association was found only with the FL, EWB, and SWB domains, as well as with the CPQ as a whole. The association between family affluence and ICON sum score was not significant (results are not shown). Girls expressed worse FL and EWB domains of OHRQoL compared with boys, while age did not significantly influence OHRQoL.

### 3.3. Path Analysis of the Associations

**Figure 1:**
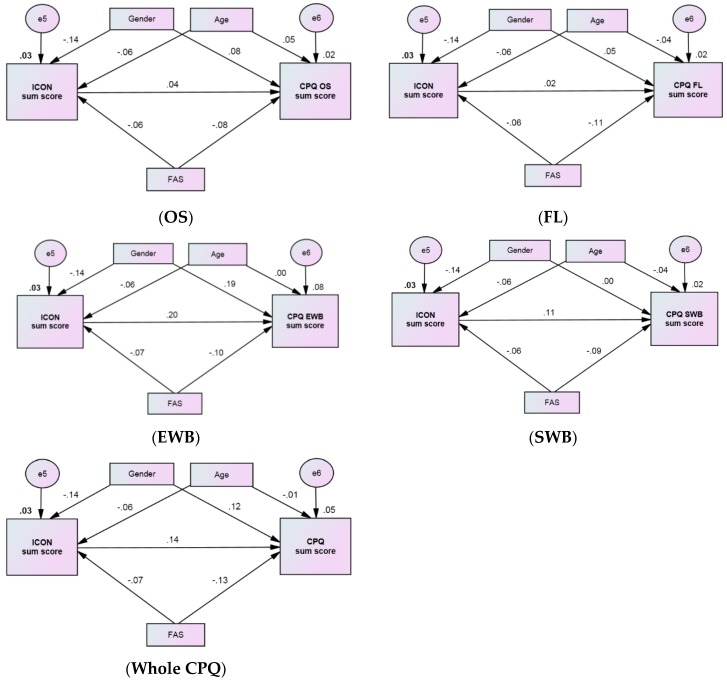
Path diagrams representing the strength of association among FAS, ICON, and the sum scores of the CPQ by CPQ domains (oral symptoms (OS), functional limitations (FL), emotional well-being (EWB), and social well-being (SWB)) and the CPQ as a whole, adjusted by gender and age.

Path analysis models were used to examine the relationships between FAS, ICON, and each of the CPQ domains or the CPQ as a whole. Additionally, gender and age effects were included in the models (Figure 1).

The statistics used to assess the fit of the models are shown in Table 4. The χ^2^ test was non-significant (*p* = 0.089 or *p* = 0.090) and the RMSEA was 0.026 (less than 0.05), indicating that the model and data were consistent. The additional fit statistics, CFI and IFI, for all the domains were greater than 0.90, which indicates a good fit of the data. The best fitness was observed for the EWB domain (CFI = 0.971 and IFI = 0.973). The squared multiple correlations had a minimal value of 0.016 for the OS domain, and a maximal value 0.080 for the EWB domain, i.e., 1.6–8.0% of all variance in the domain sum score can be explained by the variables included in the path models.

The strengths of the influences of gender, age group, and FAS group variables on the ICON sum score was almost the same among the path models of the CPQ domains. Compared to boys, girls had a significantly lower ICON sum score (*β* = −0.14; *p* < 0.001). Older adolescents and children of more affluent families also had low ICON sum scores, but this effect was either very weak or statistically insignificant.

A significant effect of ICON on the sum score of the CPQ was revealed only for the EWB (*β* = 0.20; *p* < 0.001) and SWB (*β* = 0.11; *p* < 0.001) domains. The effect of FAS on the sum score of the CPQ was significant in all domains, although its value varied between domains. The weakest association (*β* = −0.08; *p* < 0.01) was observed for the OS domain (in the case of regression analysis, this relationship was insignificant), and the strongest association (*β* = −0.11; *p* < 0.001) was observed for the FL domain. Age had no significant effect on the sum score of the CPQ in any domain, while girls had a higher sum score for the EWB domain than boys (*β* = 0.19; *p* < 0.001). As can be seen from the path models, FAS, gender, and age may also contribute to the sum scores of the CPQ indirectly via their relationships with ICON. Although the indirect effects were small, the total effect of FAS on the sum score of the CPQ became more negative. Similarly, the gender gap with regard to the total effect slightly decreased. Table 5 shows the standardized direct, indirect, and total effects of these variables on the sum score of the CPQ.

## 4. Discussion

The models examined in the current study provided a reasonable fit for the data, indicating that they effectively identified the impact of family affluence on OHRQoL among adolescents in Lithuania. All models based on the correlation analysis, negative binomial regression analysis, and path analysis showed evidence that higher family socioeconomic status has a direct positive impact on adolescent OHRQoL rather than an indirect impact via malocclusion that certainly provides a negative influence on OHRQoL.

This conclusion was reached in response to several questions. Firstly, the relationship between malocclusion and OHRQoL was assessed. Although several hypotheses have been proposed and tested, there is still no generally accepted opinion among researchers [35]. One of the causes for this might be the use of an inappropriate model (analysis method) in previous studies, since this association is much more sophisticated than a simple linear one. It is possible that, for this reason, some population-based studies reported no significant relationship between the CPQ sum score and malocclusion [14,36]. However, recent systematic reviews and meta-analyses of the literature have claimed that there is evidence for a clear negative influence of malocclusion on OHRQoL [5]. They showed also that the strength of the association differs between the OHRQoL domains. In most cases, a negative impact of malocclusion on the social and emotional domains of OHRQoL has been reported [4,5,6]. The results of the present study and our recent study [37] support this, agreeing with findings of the studies that affirmed a significant relationship between the severity of malocclusion and sum scores of the EWB and SWB domains [38,39,40,41]. The reasons for this finding is that social life and emotional perceptions play important roles in adolescents’ values. For example, visible orthodontic traits can be particularly relevant for adolescents, who can then become the victims of teasing and bullying [42,43].

The association between SES and OHRQoL seems to be commonly questioned in this field of research. We also hypothesized that there is a direct association between higher family affluence and better adolescent OHRQoL, e.g., a lower CPQ sum score. This hypothesis was confirmed by both NBR and path analyses. The link was significant for all CPQ domains but, according to the NBR analysis, the highest impact of family affluence was found in the FL, EWB, and SWB domains. Many researchers have also noted that individuals from more affluent families have significantly better OHRQoL than their peers from less affluent families [16,44,45,46,47,48]. There is evidence that children living in more affluent families are more likely to benefit from good oral hygiene, regularly visit a dentist, and be better educated in healthy lifestyle skills, thus resulting in better OHRQoL [5,49,50,51,52]. Similarly, in a study of 12–15-year-old orphans in India, Kumar et al. found that children without parents presented poorer scores for OHRQoL compared with those that had parents [53].

An indirect effect of family affluence on OHRQoL across all aspects of orthodontic status was relatively small, because a negligible relationship between malocclusion and family affluence was found. The literature contains a few studies that have investigated the impact of social variables on malocclusion prevalence among children and adolescents. Among these, several studies described severe malocclusions in lower SES adolescents undergoing orthodontic treatment [54] or a higher prevalence of malocclusion among adolescents residing in less affluent districts [55]. An inverse association, in which high SES children showed a higher prevalence of malocclusion, was revealed by Normando et al. in a study of primary dentition in children from the Brazilian Amazon [56]. Moreover, household social class was considered to be a predictor of the orthodontic treatment outcome [57]. In contrast to these studies, the present findings corroborate the results of other studies which showed that the distribution of the type and grading of the treatment need is similar throughout the different social classes [58,59].

In the present study, OHRQoL was investigated throughout the full adolescence period (11–18 years). Hence, we were able to regard adolescent age as a predictor of malocclusion and OHRQoL. The path analysis showed a moderate effect of age on the severity of malocclusion, indicating a lower ICON sum score as adolescent age increased. Several studies on malocclusion in the Lithuanian adolescent population have shown similar results [3]. However, the present analysis did not indicate any significant relationship between age and OHRQoL. The available research data on this relationship is controversial [7].

Adolescent gender appeared to be a more important factor in the prevalence of malocclusion and in the assessment of OHRQoL. The comparison of genders in the path analysis showed that the ICON sum score was lower among girls, which means that the need for orthodontic treatment was more prevalent among boys. In contrast, the literature shows that the demand for orthodontic treatment is higher among girls than among boys [60]. The gender difference in seeking of orthodontic treatment appears to be related to differences in perceived health and the value of oral health among boys and girls [51]. Our research showed that girls were more likely to experience issues related to emotional well-being (EWB domain). Similar observations have been described in other studies [40,61,62,63], although some others have not found that such a relationship exists [7,50].

The present study is one of the first studies on OHRQoL in Lithuanian adolescents [24,37]. However, methodological procedures were used to increase the power of the study, such as the validation of the CPQ instrument [24,25], obtaining a representative and population-based sample, performing a pilot study, and standardized examiner. The use of path analysis can also be considered a positive aspect due to its usefulness in the interpretation of relationships among the variables.

The main limitations of the present study are the cross-sectional design, which did not enable a causal relationship between the variables to be established. Moreover, the ICON was developed for permanent teeth and so has a tendency to be oversensitive during the mixed dentition period, possibly confounding the results in the group of adolescents aged 11–14 years [27]. In addition, a possible weakness of this study may be related to the use of the CPQ, which is a generic measure of OHRQoL, so some of its items could not address aspects specifically related to malocclusion. Regardless, the present findings highlight the impact of social factors (namely, family affluence) on adolescent OHRQoL and can assist in the establishment of more effective actions in orthodontic practice and public health.

## 5. Conclusions

This study evidences the family affluence based inequality in OHRQoL among Lithuanian adolescents. All four domains of OHRQoL were significantly associated with family affluence, indicating higher OHRQoL among adolescents who live in more affluent families. Meanwhile, the severity of malocclusion predetermined by ICON had a significant impact only on the emotional and social well-being domains of OHRQoL. These findings demonstrate the need to consider the clinical and socio-environmental factors in planning strategies for the oral health of adolescents.

## Figures and Tables

**Table 1:** Descriptive summary of study participant characteristics for all variables analyzed.

Characteristic	*n*	(%)	Mean	(SD)
*Age*	881		15.55	(1.51)
11–14 years	177	(20.1)		
15–16 years	413	(46.9)		
17–18 years	291	(33.0)		
*Gender*				
Boys/Age	360	(40.9)	15.55	(1.51)
Girls/Age	521	(59.1)	15.55	(1.51)
*Family affluence*				
Low	110	(12.5)		
Medium	348	(39.5)		
High	423	(48.0)		
*ICON sum score*	881		39.07	(21.03)
≤43	600	(68.1)		
>43	281	(31.9)		
*Whole CPQ sum score: mean (SD)*	881		10.49	(10.48)
OS	881		4.00	(2.96)
FL	881		1.94	(2.94)
EWB	881		3.30	(4.93)
SWB	881		1.25	(2.93)
*Whole CPQ sum score: median (IQR)*	881		7	(3; 14)
OS	881		3	(2; 6)
FL	881		1	(1; 3)
EWB	881		2	(0; 5)
SWB	881		0	(0; 1)

**Table 2:** Spearman’s correlation coefficients (rho) between Child Perceptions Questionnaire (CPQ), Index of Complexity, Outcome, and Need (ICON), and Family Affluence Scale (FAS) sum scores.

Sum Scores	Whole CPQ	OS	FL	EWB	SWB	FAS
ICON	0.13 **	0.04	0.02	0.18 **	0.11 **	−0.02
Whole CPQ		X	X	X	X	−0.12 **
OS			0.39 **	0.39 **	0.31 **	−0.09 *
FL				0.48 **	0.44 **	−0.08 *
EWB					0.57 **	−0.11 **
SWB						−0.05

X—the correlation does not make sense. * *p* < 0.05; ** *p* < 0.01.

**Table 3:** Association of CPQ and its four domains’ sum scores with gender, age, severity of malocclusion, and family affluence: results as determined by the multivariate Negative Binomial Regression (NBR) analysis.

Variable	RSSM (95% CI)				
	OS	FL	EWB	SWB	Whole CPQ
*Gender*					
Boys (ref.)	1.00	1.00	1.00	1.00	1.00
Girls	1.10 (0.9–1.28)	**1.19** * (1.00–1.41)	**1.79** *** (1.53–2.10)	1.07 (0.89–1.29)	**1.29** *** (1.11–1.49)
*Age*					
11–14 years (ref.)	1.00	1.00	1.00	1.00	1.00
15–16 years	1.10 (0.90–1.34)	1.10 (0.89–1.38)	0.94 (0.77–1.16)	1.12 (0.88–1.43)	1.05 (0.87–1.27)
17–18 years	1.16 (0.94–1.44)	0.97 (0.76–1.22)	1.03 (0.83–1.28)	1.03 (0.79–1.33)	1.06 (0.87–1.30)
*Severity of malocclusion*					
ICON sum score ≤43 (ref.)	1.00	1.00	1.00	1.00	1.00
ICON sum score >43	1.02 (0.87–1.19)	0.96 (0.81–1.15)	**1.61** *** (1.37–1.89)	**1.62** *** (1.35–1.95)	**1.24** ** (1.07–1.43)
*Family affluence*					
Low (ref.)	1.00	1.00	1.00	1.00	1.00
Medium	0.93 (0.73–1.18)	**0.77** * (0.59–0.99)	**0.78** * (0.61–1.00)	**0.70** * (0.53–0.92)	0.82 (0.66–1.03)
High	0.89 (0.70–1.12)	**0.69** ** (0.54–0.89)	**0.75** * (0.59–0.95)	**0.57** *** (0.44–0.75)	**0.76** * (0.61–0.95)

RSSM—Ratio of Sum Score Means; ref.—reference group. * *p* < 0.05; ** *p* < 0.01. *** *p* < 0.001.

**Table 4:** Squared Multiple Correlations and Model Goodness-of-Fit Statistics for the four domains and the CPQ as a whole.

Characteristics	Estimates				
	OS	FL	EWB	SWB	Whole CPQ
**Goodness-of-fit statistics:**					
χ^2^	6.524	6.504	6.508	6.488	6.526
Degree of freedom	3	3	3	3	3
χ^2^ /Degree of freedom	2.175	2.168	2.169	2.164	2.175
*p*	0.089	0.090	0.089	0.090	0.089
RMSEA (90% CI)	0.026 (0–0.054)	0.026 (0–0.054)	0.026 (0–0.054)	0.026 (0–0.054)	0.026 (0–0.054)
CFI	0.912	0.913	0.971	0.916	0.957
IFI	0.932	0.933	0.973	0.935	0.963
**Squared multiple correlations:**					
CPQx sum score	0.016	0.017	0.080	0.024	0.050

CPQx—OS, FL, EWB, SWB or whole CPQ.

**Table 5:** Standardized direct, indirect and total effects of ICON, gender, age, and family affluence on the oral-health-related quality of life (OHRQoL), assessed in four domains and for the CPQ as a whole.

Path	Way of Effect	Standardized Regression Weights				
		OS	FL	EWB	SWB	Whole CPQ
ICON sum score	Direct effect	0.041	0.018	0.202 ***	0.112 ***	0.142 ***
Gender	Direct effect	0.081 **	0.045	0.190 ***	0.001	0.123 ***
	Indirect effect	−0.006	−0.003	−0.028	−0.028	−0.020
	Total effect	0.075 *	0.042	0.162 ***	−0.019	0.103 ***
Age	Direct effect	0.050 *	−0.037	−0.001	−0.040	−0.009
	Indirect effect	−0.002	−0.001	−0.013	−0.007	−0.009
	Total effect	0.048	−0.038	−0.014	−0.047	−0.018
FAS	Direct effect	−0.080 **	−0.113 ***	−0.101 ***	−0.092 ***	−0.129 ***
	Indirect effect	−0.003	−0.001	−0.014	−0.007	−0.010
	Total effect	−0.083 **	−0.114 **	−0.115 **	−0.097 ***	−0.139

CPQx—OS, FL, EWB, SWB or whole CPQ. * *p* < 0.05; ** *p* < 0.01. *** *p* < 0.001.

## Supplementary Materials

The following are available online at https://www.mdpi.com/1660-4601/16/12/2106/s1, File S1. Questionnaire for schoolchildren: CPQ and FAS, (PDF 270 kb). File S2. Record form for oral health examination: ICON, (PDF 216 kb)

## Abbreviations

The following abbreviations are used in this manuscript:

AC	Aesthetic Component
CI	Confidence Interval
CPQ	Child Perceptions Questionnaire
EWB	Emotional Well-Being
FAS	Family Affluence Scale
FL	Functional Limitations
ICON	Index of Complexity Outcome and Need
NBR	Negative Binomial Regression
OHRQoL	Oral Health-Related Quality of Life
OS	Oral Symptoms
RSSM	Ratio of Sum Score Means
QoL	Quality of Life
SD	Standard Deviation
SES	Socio-Economic Status
SWB	Social Well-Being
