# Supplementary material for: Family Affluence Based Inequality in Oral Health-Related Quality of Life in a Population of Lithuanian Adolescents
Source: Int J Environ Res Public Health. 2019 Jun 14;16(12):2106. doi: 10.3390/ijerph16122106 (PMC6616952; doi:10.3390/ijerph16122106)
Supplement: Supplementary file 1 [file ijerph-16-02106-s001.zip › File S1-Family affluence and OHRQoL.pdf]

THE STUDY ON
CHILDREN'S ORAL HEALTH
RELATED QUALITY OF LIFE

QUESTIONNAIRE FOR
SCHOOLCHILDREN

EXTRACTION
CHILD PERCEPTIONS QUESTIONNAIRE (CPQ)
&
FAMILY AFFLUENCE SCALE (FAS)
ONLY

Dear Student,

Thanks for agreeing to participate in our study. By answering the questions you will help us to learn more about children's oral health problems and experiences. This will allow exploring deeper children's dental and other mouth organ diseases, to reveal their reasons, to pick the best treatment. It will be useful, because healthy teeth and mouth is full with good for your health and happiness.

How to fill out the questionnaire? Read each question carefully. Put cross "X" in the box, which is near or below the answer that is best for you. For each question, select <u>only one</u> answer, because otherwise we will not be able to count your answers. If it is difficult to choose a single answer, think about answer that at the moment is the most precise. In other cases, write your reply above the points in the selected location.

Very please reply to the questions on your own. Don't talk to anyone about the questions when you are answering them. Your answers are private. We promise that no one at the school and the parents (guardians) will know your answers. After filling out the questionnaire, put it in an envelope, seal and give it to the dentists visiting your school. Don't write your name neither on the questionnaire nor the envelope.

Thanks in advance for honesty and sincerity

The study is conducted by team of researchers from
Lithuanian University of Health Sciences
Clinic of Orthodontics and
Clinic of Preventive and Children Odontology

1. **Today's date:** _________ / __________ / 201____

DAY MONTH YEAR

Initially, a few questions about you

2. **Date of your birth:** _________ / __________ / ________

DAY MONTH YEAR

3. **Are you a boy or a girl?**

Cross (X) an appropriate box

1❑ Boy

2❑ Girl

Questions about your oral health

1. Over the last 3 months, have you felt any pain in the following mouth organs?
Select one box in each line

	1<br>*Never*	2<br>*Once or twice*	3<br>*Sometimes*	4<br>*Often*	5<br>*Almost every day*
a) Teeth	❑	❑	❑	❑	❑
b) Lips	❑	❑	❑	❑	❑
c) Gums	❑	❑	❑	❑	❑
d) The lining of the mouth organ	❑	❑	❑	❑	❑
e) Jaws and joints	❑	❑	❑	❑	❑

2. Over the past 3 months, how often have you had the following?
Select one box in each line

	1<br>*Never*	2<br>*Once or twice*	3<br>*Sometimes*	4<br>*Often*	5<br>*Almost every day*
a) Bleeding gums while brushing teeth	❑	❑	❑	❑	❑
b) Had sores and wounds in your mouth	❑	❑	❑	❑	❑
c) Bad breath	❑	❑	❑	❑	❑
d) Food stuck in or between the teeth	❑	❑	❑	❑	❑
e) Food stuck in the top of your mouth	❑	❑	❑	❑	❑

Questions about limitations related to your oral health

3. In the past 3 months, how often has this happened because of your teeth and oral health?

Select one box in each line

	1 *Never*	2 *Once or twice*	3 *Sometimes*	4 *Often*	5 *Almost every day*
a) Difficult to bite or chew hard solid food like apples or meat	❑	❑	❑	❑	❑
b) Difficult to open your mouth wide	❑	❑	❑	❑	❑
c) Difficult to say any words	❑	❑	❑	❑	❑
d) Difficult to eat foods you would like to eat	❑	❑	❑	❑	❑
e) Difficult to drink with a straw	❑	❑	❑	❑	❑
f) Difficult to drink or eat hot or cold foods	❑	❑	❑	❑	❑

4. In the past 3 months, how often has this happened because of your teeth and oral conditions?

Select one box in each line. ***If this was not associated with dental or oral health, note the answer 'Never'.***

	1 *Never*	2 *Once or twice*	3 *Sometimes*	4 *Often*	5 *Almost every day*
a) Breathed through your mouth	❑	❑	❑	❑	❑
b) Taken longer than others to eat meal	❑	❑	❑	❑	❑
c) Had trouble sleeping	❑	❑	❑	❑	❑

Questions about your feelings and emotions caused by oral health

5. In the past 3 months, how often have you had these feelings and emotions because of your teeth and oral health?
Select one box in each line. ***If this was not associated with dental or oral health, note the answer 'Never'.***

		1<br>*Never*	2<br>*Once or twice*	3<br>*Sometimes*	4<br>*Often*	5<br>*Almost every day*
a)	You felt irritable or frustrated	❑	❑	❑	❑	❑
b)	You felt unsure of yourself	❑	❑	❑	❑	❑
c)	You felt shy or embarrassed	❑	❑	❑	❑	❑
d)	You concerned that other people have bad thoughts about your teeth or mouth	❑	❑	❑	❑	❑
e)	You worried that your teeth or mouth are not as good-looking as others	❑	❑	❑	❑	❑
f)	You have been upset because of your teeth or mouth	❑	❑	❑	❑	❑
g)	You felt nervous or afraid because of your teeth or mouth	❑	❑	❑	❑	❑
h)	You worried that your teeth or mouth are not as healthy as others	❑	❑	❑	❑	❑
i)	You worried that your teeth or mouth appear different than other people	❑	❑	❑	❑	❑

Questions about school, friends and leisure time

6. In the <u>past 3 months</u>, how often have you had these experiences because of your teeth and oral health?
Select one box in each line. ***If this was not associated with dental or oral condition, note the answer 'Never'.***

	1<br>*Never*	2<br>*Once or twice*	3<br>*Sometimes*	4<br>*Often*	5<br>*Almost every day*
a) Avoided to go to school because of your teeth or mouth	❑	❑	❑	❑	❑
b) Could not con-centrate at school	❑	❑	❑	❑	❑
c) Could not concen-trate on homework	❑	❑	❑	❑	❑
d) Avoided to speak or read aloud in classroom	❑	❑	❑	❑	❑
e) Avoided taking part in common activities (sports, choir, get together, etc.)	❑	❑	❑	❑	❑
f) You not wonted to talk to other people	❑	❑	❑	❑	❑
g) Avoided smiling or laughing when around other people	❑	❑	❑	❑	❑
h) Generally speaking, you avoided being with friends	❑	❑	❑	❑	❑
i) You argued with other children or your family	❑	❑	❑	❑	❑
j) It was difficult to play the wind musical instrument (if you play)	❑	❑	❑	❑	❑

7. In the <u>past 3 months</u>, how often have other children had the following because of your teeth and oral health?

Select one box in each line. ***If this was not associated with dental or oral condition, note the answer 'Never'.***

		1<br>*Never*	2<br>*Once or twice*	3<br>*Sometimes*	4<br>*Often*	5<br>*Almost every day*
a)	Other children teased you or called names	❑	❑	❑	❑	❑
b)	Other children made you feel left out	❑	❑	❑	❑	❑
c)	Other children asked you questions about your teeth and mouth	❑	❑	❑	❑	❑

Questions about your parents and family

1. How many computers does your family own?

1 ❑ None
2 ❑ One
3 ❑ Two
4 ❑ Three or more

2. Does your family own a car, van or truck?

1 ❑ No
2 ❑ Yes, one
3 ❑ Yes, two or more

3. During the past 12 months, how many times did you travel away on holiday (vacation) with your family?

1 ❑ Not at all
2 ❑ Once
3 ❑ Twice
4 ❑ More than twice

4. Do you have your own bedroom for yourself?

1 ❑ No
2 ❑ Yes

Thank you for your honest answers.
Please check if you have answered to all questions.
