# Supplementary material for: Family Affluence Based Inequality in Oral Health-Related Quality of Life in a Population of Lithuanian Adolescents
Source: Int J Environ Res Public Health. 2019 Jun 14;16(12):2106. doi: 10.3390/ijerph16122106 (PMC6616952; doi:10.3390/ijerph16122106)
Supplement: Supplementary file 1 [file ijerph-16-02106-s001.zip › File S2-Family affluence and OHRQoL.pdf]

THE STUDY ON
CHILDREN'S ORAL HEALTH
RELATED QUALITY OF LIFE

RECORD FORM FOR
ORAL HEALTH EXAMINATION

EXTRACTION
INDEX OF COMPLEXITY, OUTCOME AND NEED (ICON)
ONLY

Aesthetic component (AC) of the ICON

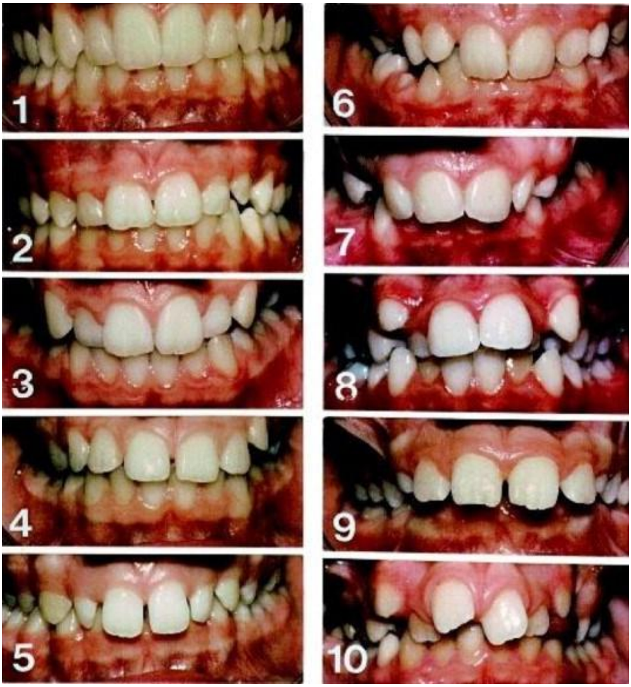

Figure 1. Aesthetic Assessment 10 photographs showing different level of dental attractiveness on a scale 1–10 [27]

AC **Aesthetic assessment (1.....10)**

....

Dental component of the ICON

ICON1 A. Upper arch crowding

- 0❑ ≤ 2 mm
- 1❑ 2.1 to 5 mm
- 2❑ 5.1 to 9 mm
- 3❑ 9.1 to 13 mm
- 4❑ 13.1 to 17 mm
- 5❑ ≥ 17.1 mm

B. Upper arch spacing

- 0❑ ≤ 2 mm
- 1❑ 2.1 to 5 mm
- 2❑ 5.1 to 9 mm
- 3❑ 9.1 to 13 mm
- 4❑ ≥ 13.1 mm
- 5❑ Impacted teeth

ICON2. Crossbite

- 0❑ No
- 1❑ Yes

ICON3 A. Incisor open bite

- 0❑ Edge to edge
- 1❑ < 1 mm
- 2❑ 1.1 to 2 mm
- 3❑ 2.1 to 4 mm
- 4❑ ≥ 4.1 mm

B. Incisor overbite

- 0❑ <1/3 lower incisor coverage
- 1❑ 1/3 to 2/3 coverage
- 2❑ 2/3 up to full covered
- 3❑ Full lower incisor coverage

ICON4 The fit of the upper and lower posterior teeth in an antero -posterior perspective:

Right side		Left side
0❑	Cusp to cusp embrasure only (Class I, II, III)	0❑
1❑	Any cusp relationship up to, but not including cusp to cusp	1❑
2❑	Cusp to cusp	2❑

Table 1. *ICON Scoring Matrix and Calculation of Scores*

**Variable**	**Component**	**ICON scores**						**Weight**
		**0**	**1**	**2**	**3**	**4**	**5**	
AC	***Aesthetic assessment***	1–10						7
ICON1A	***The first dental component:*** Upper arch crowding	≤ 2 mm	2.1 to5 mm	5.1 to 9 mm	9.1 to 13 mm	13.1 to 17 mm	≥ 17.1 mm	5
ICON1B	Upper arch spacing	≤ 2 mm	2.1 to 5 mm	5.1 to 9 mm	9.1 to 13 mm	≥ 13.1 mm	Impacted teeth	5
ICON2	***The second dental component:*** Crossbite	No crossbite	Crossbite present					5
ICON3A	***The third dental component:*** Incisor open bite	Edge to edge	< 1 mm	1.1 to 2 mm	2.1 to 4 mm	≥ 4.1 mm		4
ICON3B	Incisor overbite	< 1/3 lower incisor coverage	1/3 to 2/3 coverage	2/3 up to fully covered	Fully covered			4
ICON4R	***The fourth dental component:*** The fit of the upper and lower posterior teeth in an antero-posterior perspective: Right side	Cusp to embrasure only: Class I, II, III	Any cusp relation up to, but not including cusp to cusp	Cusp to cusp				3
ICON4L	Left side							
**ICON sum score = AC × 7 + ICON1A × 5 + ICON1B × 5 + ICON2 × 5 + ICON3A × 4 + ICON3B × 4 + ICON4R × 3+ ICON4L × 3**								
